# Enhanced construction of gene regulatory networks using hub gene information

**DOI:** 10.1186/s12859-017-1576-1

**Published:** 2017-03-23

**Authors:** Donghyeon Yu, Johan Lim, Xinlei Wang, Faming Liang, Guanghua Xiao

**Affiliations:** 1 Department of Statistics, Inha University, Incheon, Korea; 2 Department of Statistics, Seoul National University, Seoul, Korea; 3 Department of Statistical Science, Southern Methodist University, 6425 Boaz Lane, Dallas, TX 75205 USA; 4 Department of Biostatistics, University of Florida, 2004 Mowry Road, Gainesville, FL 32611 USA; 5 Department of Clinical Sciences, University of Texas Southwestern Medical Center, 5323 Harry Hines Blvd., Dallas, TX 75390 USA

**Keywords:** Gene regulatory network, Hub gene, Partial correlation, Sparse partial correlation estimation, *Escherichia coli*, Lung cancer

## Abstract

**Background:**

Gene regulatory networks reveal how genes work together to carry out their biological functions. Reconstructions of gene networks from gene expression data greatly facilitate our understanding of underlying biological mechanisms and provide new opportunities for biomarker and drug discoveries. In gene networks, a gene that has many interactions with other genes is called a hub gene, which usually plays an essential role in gene regulation and biological processes. In this study, we developed a method for reconstructing gene networks using a partial correlation-based approach that incorporates prior information about hub genes. Through simulation studies and two real-data examples, we compare the performance in estimating the network structures between the existing methods and the proposed method.

**Results:**

In simulation studies, we show that the proposed strategy reduces errors in estimating network structures compared to the existing methods. When applied to *Escherichia coli*, the regulation network constructed by our proposed ESPACE method is more consistent with current biological knowledge than the SPACE method. Furthermore, application of the proposed method in lung cancer has identified hub genes whose mRNA expression predicts cancer progress and patient response to treatment.

**Conclusions:**

We have demonstrated that incorporating hub gene information in estimating network structures can improve the performance of the existing methods.

## Background

A gene regulatory network (GRN) describes interactions and regulatory relationships among genes. It provides a systematic understanding of the molecular mechanisms underlying biological processes by revealing how genes work together to form modules that carry out cell functions [1–4]. In addition, the visualization of genetic dependencies through the GRN facilitates the systematic interpretation and comprehension of analysis results from genome-wide studies using high-throughput data. GRNs have proven valuable in a variety of contexts, including identifying druggable targets [5], detecting driver genes in diseases [6], and even optimizing prognostic and predictive signatures [7].

Gene expression microarrays monitor the transcription activities of thousands of genes simultaneously, which provides a great opportunity to study the “relationships” among genes on a large scale. However, challenges lie in constructing large-scale GRNs from gene expression microarray data due to the small sample sizes of microarray studies and the extremely large solution space. Computational techniques and algorithms have been proposed to reconstruct GRNs from gene expression data, including probability-based approaches such as Bayesian networks [8–12], correlation-based approaches [13], likelihood-based approaches [14–16], partial-correlation-based approaches [17, 18], and information-theory-based approaches [19–22]. The existing methods are briefly reviewed in the Methods Section. Readers can also refer to Bansal et al. [23] and Allan et al. [24] for a more detailed review of network construction methods.

The sparse partial correlation estimation (SPACE) method, proposed by Peng et al. [18], considers a penalized regression approach to estimate edges in the GRN, which utilizes the sparse feature of the GRN. Comparative studies have shown that the SPACE method performs well in estimating sparse networks with high accuracy [24]. Peng et al. [18] also showed that the method was able to identify functional relevant molecular networks. In addition, recent studies of network analysis have revealed its advantage in detecting genes or modules associated with phenotypes [25–27].

In gene networks, genes that have many interactions with other genes are defined as hub gens. Because of these interactions, hub genes usually play an important role in a biological system. For example, transcription factor (TF), a protein that binds to specific DNA sequences, can regulate a given set of genes. In humans, approximately 10% of genes in the genome code for around 2600 TFs [28]. The combinatorial human TFs account for most of the regulation activities in the human genome, especially during the development stage. As a result, the genes that code TFs, called TF-encoding genes, are usually regarded as hub genes. Furthermore, in cancer research, cancer genes (oncogenes or tumor suppressor genes) take part in tumor genesis and are likely to be hub genes in the genetic networks of tumors [29, 30]. Through decades of biological studies, knowledge on important genes (such as TFs or cancer genes) has been accumulated. Our hypothesis is that incorporating prior knowledge about hub genes can improve accuracy in estimating the gene network structure. It is worth noting that there is a reweighted *ℓ*_1_ regularization method [31] that repeatedly estimates the structures and modifies the weights of the penalties by using the information on degrees from the previous estimation to encourage the appearance of the hubs. This method does not use the prior information obtainable from the other resources while our method uses additional information not contained in the observed dataset.

To explicitly account for the information on hub genes, we propose an extension of the SPACE method, which introduces an additional tuning parameter to open up the possibility of reducing penalization and increasing the likelihood of selecting the edges connected to such genes. We numerically show that the proposed method reduces errors in estimating network structures. Although we focus on extending the SPACE method in this paper, the idea can also be applied to penalized likelihood methods as well as to other penalized regression methods. Note that there is no rigorous definition of a hub in the context of a network; the definition of a hub varies depending on the sparsity of the network. For sparse protein networks, a hub is defined in [32] as a protein whose degree lies over the 0.95 quantile of the degree distribution or in [33] and [7] as a protein whose degree is greater than 7. In this paper, we conservatively define a hub as a node whose degree is both greater than 7 and above the 0.95 quantile of the degree distribution, because most nodes in sparse networks have relatively small degrees between 0 and 3.

In this study, we briefly introduce seven existing methods, including the SPACE and the graphical lasso, and propose the extended SPACE (ESPACE) method to incorporate the biological knowledge about important genes, i.e. network hubs. Moreover, it is worth noting that the ESPACE only incorporates the previously known biological information not contained in the observed dataset compared to the other existing methods. Through simulation studies, we show that the proposed approach reduces error in estimating the network structures compared to the seven other existing methods that we reviewed in the “Methods” section. Finally, we demonstrate the improvement of the ESPACE method compared to the SPACE method with two real-data examples.

## Methods

### Review of existing methods

Here, we briefly review the existing methods; the GeneNet [34], the NS [17], the GLASSO [15], the GLASSO-SF [31], the PCACMI [21], the CMI2NI [22], and the SPACE [18]. Let $X_{i}^{k}$ be the expression level of the *i*th gene of the *k*th array for *i*=1,2,…,*p* and *k*=1,2,…,*n*. Let $\mathbf {X}_{i} = \left (X_{i}^{1}, X_{i}^{2}, \ldots, X_{i}^{n}\right)^{T}$ so that observed gene expression data can be denoted by an *n*×*p* matrix **X**=(**X**_1_;**X**_2_;…;**X**_*p*_) whose rows and columns denote arrays and genes, respectively. Suppose row vectors $\mathbf {X}^{k}=\left (X_{1}^{k},X_{2}^{k},\ldots,X_{p}^{k}\right)$ for *k*=1,2,…,*n* are independently and identically distributed random vectors from the multivariate normal distribution with mean **0** and covariance matrix *Σ*. We assume that *Σ* is positive definite, and let *Ω*≡*Σ* ^−1^=(*ω*_*ij*_)_1≤*i*,*j*≤*p*_ be the inverse of the covariance matrix *Σ*, which is referred to as a concentration matrix or a precision matrix.

#### GeneNet

Schäfer and Strimmer [34] propose the linear shrinkage estimator for a covariance matrix and the Gaussian graphical model (GGM) selection based on the partial correlation obtained from their shrinkage estimator. With multiple testing procedure using the local false discovery rate [35], the GGM selection controls the false discovery rate under a pre-determined level *α*. Since Schäfer and Strimmer [34] provide their GGM selection procedure in the R package GeneNet, we denote their GGM selection procedure as GeneNet in this paper. To be specific, one of the most commonly used linear shrinkage estimators *S* ^∗^ for the covariance matrix *Σ* is

$$  S^{*} = \lambda^{*} T + (1-\lambda^{*}) S,  $$

where *S*=(*s*_*ij*_)_1≤*i*,*j*≤*p*_ is the sample covariance matrix, *T*=diag(*s*_11_,*s*_22_,…,*s*_*pp*_) is the shrinkage target matrix, and $\lambda ^{*} = {\sum \nolimits }_{i\neq j} \widehat {\text {Var}} (s_{ij}) / \left ({\sum \nolimits }_{i\neq j} s_{ij}^{2}\right)$ is the optimal shrinkage intensity. With this estimator *S* ^∗^, the matrix of the partial correlations $P = (\hat {\rho }^{ij})_{1 \le i,j \le p}$ is defined as $\hat {\rho }^{ij} = -\hat {\omega }_{ij} / \sqrt {\hat {\omega }_{ii} \hat {\omega }_{jj}}$, where $\hat {\Omega } = (\hat {\omega }_{ij})_{1 \le i,j \le p} = \left (S^{*}\right)^{-1}$.

To identify the significant edges, Schäfer and Strimmer [34] suppose the distribution of the partial correlations as the mixture

$$f(\rho) = \eta_{0} f_{0} (\rho,\nu) + (1-\eta_{0}) f_{1}(\rho), $$

where *f*_0_ is the null distribution, *f*_1_ is the alternative distribution corresponding to the true edges, and *η*_0_ is the unknown mixing parameter. Using the algorithm in [35], GeneNet identifies significant edges that have the local false positive rate

$$\text{fdr}(\rho) = \frac{\hat{\eta}_{0} f_{0} (\rho, \hat{\nu})}{\hat{f}(\rho)} $$

smaller than the pre-determined level *α*, where *f*_0_(*ρ*,*ν*)=|*ρ*|Be(*ρ* ^2^;0.5,(*ν*−1)/2), Be(*x*;*a*,*b*) is the density of the Beta distribution and *ν* is the reciprocal variance of the null *ρ*.

#### Neighborhood selection (NS)

Meinshausen and Bühlmann [17] propose the neighborhood selection (NS) method, which separately solves the lasso [36] problem and identifies edges with nonzero estimated regression coefficients for each node. Meinshausen and Bühlmann [17] prove that the NS method is asymptotically consistent in identifying the neighborhood of each node when the neighborhood stability condition is satisfied. Note that the neighborhood stability condition is related to the irrepresentable condition in linear model literature [37].

To be specific, for each node *i*∈*V*={1,2,…,*p*}, NS solves the following lasso problem

$$\hat{\beta}^{i,\lambda} = \operatornamewithlimits{arg\min}_{\beta\in \mathbb{R}^{p}: \beta_{i} = 0} ~\frac{1}{2} \| \mathbf{X}_{i} - \mathbf{X}\beta\|_{2}^{2} + \lambda \|\beta\|_{1}, $$

where $\|\mathbf {x}\|_{2}^{2} = {\sum \nolimits }_{i=1}^{p} x_{i}^{2}$ and $\|\mathbf {x}\|_{1} = {\sum \nolimits }_{i=1}^{p} |x_{i}|$ for $\mathbf {x} \in \mathbb {R}^{p}$. With the estimate $\hat {\beta }^{i,\lambda }$, NS identifies the neighborhood of the node *i* as $N_{i}(\lambda) = \{ k~|~ \hat {\beta }_{k}^{i,\lambda } \neq 0\}$, which defines an edge set $E_{i}^{\lambda } = \{(i,j)~|~ j \in N_{i}(\lambda)\}$. Since NS separately solves *p* lasso problems, contradictory edges may occur when we define the total edge set $E^{\lambda } = \cup _{i=1}^{p} E_{i}^{\lambda }$, i.e., $\hat {\beta }_{k}^{i,\lambda } \neq 0$ and $\hat {\beta }_{i}^{k,\lambda } = 0$. To avoid these contradictory edges, NS suggests two types of edge sets *E* ^*λ*,∧^ and *E* ^*λ*,∨^ defined as follows:

$$\begin{array}{*{20}l} E^{\lambda,\wedge} = \left\{(i,j)~|~ i \in N_{j}(\lambda) ~\text{and}~ j \in N_{i}(\lambda)\right\},\\ E^{\lambda,\vee} = \left\{(i,j)~|~ i \in N_{j}(\lambda) ~\text{or}~ j \in N_{i}(\lambda)\right\}. \end{array} $$

Meinshausen and Bühlmann [17] mentioned these two edge sets have only small differences in their experience and the differences vanish asymptotically. Meinshausen and Bühlmann [17] also propose the choice of the tuning parameter *λ*_*i*_(*α*) for the *i*th node

$$\lambda_{i}(\alpha) = \|\mathbf{X}_{i}\|_{2} \tilde{\Phi}^{-1}\left(\frac{\alpha}{2p^{2}}\right), $$

where $\tilde {\Phi } = 1 - \Phi $ and *Φ* is the distribution function of the standard normal distribution. With this choice of *λ*_*i*_(*α*) for *i*=1,2,…,*p*, the probability of falsely identifying edges in the network is bounded by the level *α*. Note that we estimate the edge set with *E* ^*λ*,∧^ and solve the lasso problems using the R package CDLasso proposed by [38] in this paper.

#### Graphical lasso (GLASSO)

Friedman et al. [15] propose the graphical lasso method that estimates a sparse inverse covariance matrix *Ω* by maximizing the *ℓ*_1_ penalized log-likelihood

1 $$  l(\Omega) = \log |\Omega| - \text{tr}(S\Omega) - \lambda \|\Omega\|_{1},  $$

where *S* is the sample covariance matrix, tr(*A*) is the trace of *A* and ∥*A*∥_1_ is the *ℓ*_1_ norm of *A* for $A \in \mathbb {R}^{p \times p}$.

To be specific, let *W* be the estimate of the covariance matrix *Σ* and consider partitioning *W* and *S*

$$ W = \left(\begin{array}{cc} W_{11} & w_{12}\\ w_{12}^{T} & w_{22} \end{array}\right),~ S = \left(\begin{array}{cc} S_{11} & s_{12}\\ s_{12}^{T} & s_{22} \end{array}\right), ~ \Omega = \left(\begin{array}{cc} \Omega_{11} & \omega_{12}\\ \omega_{12}^{T} & \omega_{22} \end{array}\right) $$

Motivated by [39], Friedman et al. [15] show that the solution $\hat {\Omega }$ of (1) is equivalent to the inverse of *W* whose partitioned entity *w*_12_ satisfies *w*_12_=*W*_11_ *β* ^∗^, where *β* ^∗^ is the solution of the lasso problem

2 $$  \min_{\beta} ~ \frac{1}{2} \left\| W^{1/2}_{11} \beta - W_{11}^{-1/2} s_{12} \right\|_{2}^{2} + \lambda \|\beta\|_{1}.  $$

Based on the above property, the graphical lasso sets the diagonal elements *w*_*ii*_=*s*_*ii*_+*ρ* and obtains the off-diagonal elements of *W* by repeatedly applying the following two steps:

1. Permuting the columns and rows to locate the target elements at the position of *w*_12_.
2. Finding the solution *w*_12_=*W*_11_ *β* ^∗^ by solving the lasso problem (2).

until convergence occurs. After finding *W*, the estimate $\hat {\Omega }$ is obtained from the relationships $\omega _{12} = - \hat {\beta } \hat {\omega }_{22}$ and $\hat {\omega }_{22} = 1/(w_{22} - w_{12}^{T}\hat {\beta })$, where $\hat {\beta } = W_{11}^{-1} w_{12}$. This graphical lasso algorithm was proposed in [15] and had its computational efficiency improved in [16] and [40]. Witten et al. [16] provide the R package glasso version 1.7.

#### GLASSO with reweighted strategy for scale-free network (GLASSO-SF)

Liu and Ihler [31] propose the reweighted *ℓ*_1_ regularization method to improve the performance of the estimation for the scale-free network whose degrees follows the power law distribution. Motivated by the fact that the existing methods work poorly for the scale-free networks, Liu and Ihler [31] consider changing the *ℓ*_1_ norm penalty in the existing methods to the power law regularization

3 $$ p_{\lambda,\gamma}(\Omega) = \lambda \sum\limits_{i=1}^{p} \log \left(\|\omega_{-i}\|_{1} + \epsilon_{i} \right) + \gamma \sum\limits_{i=1}^{p} |\omega_{ii}|,  $$

where *λ* and *γ* are nonnegative tuning parameters, *ω*_−*i*_={*ω*_*ij*_ | *j*≠*i*}, $\|\omega _{-i}\|_{1} = {\sum \nolimits }_{j\neq i} |\omega _{ij}|$, and *ε*_*i*_ is a small positive number for *i*=1,2,…,*p*. Thus, Liu and Ihler [31] consider optimizing the following objective function

4 $$ f(\Omega; \mathbf{X}, \lambda, \gamma) = L(\mathbf{X},\Omega) + u_{L} \cdot p_{\lambda,\gamma}(\Omega),  $$

where *L*(**X**,*Ω*) denotes the objective function of the existing method without its penalty terms, *u*_*L*_=1 if *L* is convex and *u*_*L*_=−1 if *L* is concave for *Ω*. Note that the choice of *L* is flexible. For instance, *L*(**X**,*Ω*) can be the log-likelihood function of *Ω* as in the graphical lasso or the squared loss function as in the NS and the SPACE. In this section, we suppose that *L* is concave for the purpose of notational simplicity.

To obtain the maximizer of *f*(*Ω*;**X**,*λ*,*γ*), Liu and Ihler [31] propose the iteratively reweighted *ℓ*_1_ regularization procedure based on the minorization-maximization (MM) algorithm [41]. The reweighted procedure iteratively solves the following problem:

5 $$   \Omega^{(k+1)} = \operatornamewithlimits{arg\max}_{\Omega}~ L(\mathbf{X}, \Omega) - \sum\limits_{i=1}^{p} \sum\limits_{j\neq i} \eta_{ij}^{(k)} |\omega_{ij}| - \gamma \sum\limits_{i=1}^{p} |\omega_{ii}|,  $$

where $\Omega ^{(k)}= \left (\omega _{ij}^{(k)}\right)$ is the estimate at the *k*th iteration, $\|\omega _{-i}^{(k)}\|_{1} = {\sum \nolimits }_{l \neq i} |\omega _{il}^{(k)}|$, and $\eta _{ij}^{(k)} = \lambda \left (1/(\|\omega _{-i}^{(k)}\|_{1}\right. + \epsilon _{i}) +\left.1/(\|\omega _{-j}^{(k)}\|_{1} + \epsilon _{j})\right)$. In practice, [31] suggest *ε*_*i*_=1, *γ*=2*λ*/*ε*_*i*_, and the initial estimate *Ω* ^(0)^=*I*_*p*_, where *I*_*p*_ is the *p*-dimensional identity matrix. Note that this reweighted strategy facilitates to estimate the hub nodes by adjusting weights in the penalty term but weights are updated by solely using the observed dataset without previously known information from other literatures.

In this paper, we consider *L*(**X**,*Ω*)= log|*Ω*|−tr(*S* *Ω*), which is the same to the component in the objective function of the GLASSO. Thus, we call this procedure as the GLASSO with a reweighted strategy for the scale-free network (GLASSO-SF). As applied in [31], we stop the reweighting iteration after 5 iterations. The R package glasso version 1.7 is used to obtain the solution of (5) at each iteration with the penalty matrix $E^{(k)} = (e_{ij}^{(k)})$, where $e_{ij}^{(k)} = \eta _{ij}^{(k)}$ for *i*≠*j* and $e_{ii}^{(k)} = 2\lambda $ for *i*=1,2,…,*p*.

#### Path consistency algorithm based on conditional mutual information (PCACMI)

Mutual information (MI) is a widely used measure of dependency between variables in information theory. MI even measures non-linear dependency between variables and can be considered as a generalization of the correlation. Several mutual information (MI) based methods have been developed such as ARACNE [20], CLR [42], and minet [43]. However, similar to the correlation, MI only measures pairwise dependency between two variables. Thus, it usually identifies many undirected interactions between variables. To resolve this difficulty, Zhang et al. [21] propose the information theoretic method for reconstruction of the gene regulatory networks based on the conditional mutual information (CMI).

To be specific, let *H*(*X*) and *H*(*X*,*Y*) be the entropy of a random variable *X* and the joint entropy of random variables *X* and *Y*, respectively. For two random variables *X* and *Y*, *H*(*X*) and *H*(*X*,*Y*) can be expressed as

$$ H(X) = E \left(-\log f_{X}(X)\right),~ H(X,Y) = E\left(-\log f_{XY}(X,Y)\right), $$

where *f*_*X*_(*x*) is the marginal probability density function (PDF) of *X* and *f*_*XY*_(*x*,*y*) is the joint PDF of *X* and *Y*. With these notations, MI is defined as

6 $$ \begin{array}{lll} I(X,Y) &=& E\left(- \text{log} \frac{f_{XY}(X,Y)}{f_{X}(X)f_{Y}(Y)}\right)\\ &=& H(X) + H(Y) - H(X,Y). \end{array}  $$

It is known that MI measures dependency between two variables that contain both directed dependency and indirected dependency through other variables. While MI can not distinguish directed and indirected dependency, CMI can measure directed dependency between two variables by conditioning on other variables. CMI for *X* and *Y* given *Z* is defined as

7 $$  I(X,Y|Z) = H(X,Z)+ H(Y,Z) - H(Z) - H(X,Y,Z).  $$

To estimate the entropies in (7), Zhang et al. [21] consider the Gaussian kernel density estimator used in [19]. Using the Gaussian kernel density estimator, MI and CMI are defined as

8 $$\begin{array}{*{20}l} \widehat{I}(X,Y) = \frac{1}{2} \log \frac{|C(X)|~|C(Y)|}{|C(X,Y)|},\\ \widehat{I}(X,Y|Z) = \frac{1}{2} \log \frac{|C(X,Z)|~|C(Y,Z)|}{|C(Z)|~|C(X,Y,Z)|}, \end{array} $$

where |*A*| is the determinant of a matrix *A*, *C*(*X*), *C*(*Y*) and *C*(*Z*) are the variances of *X*, *Y* and *Z*, respectively, and *C*(*X*,*Z*), *C*(*Y*,*Z*) and *C*(*X*,*Y*,*Z*) are the covariance matrices of (*X*,*Z*), (*Y*,*Z*) and (*X*,*Y*,*Z*), respectively.

To efficiently identify dependent pairs of variables, Zhang et al. [21] adopt the path consistency algorithm (PCA) in [44]. Thus, the authors called their procedure as PCA based on CMI (PCACMI). The PCACMI method sets *L* = 0 and calculates with L-order CMI, which is equivalent to MI if *L*=0. Then, PCACMI removes the pairs of variables such that the maximal CMI of two variables given *L*+1 adjacent variables is less than a given threshold *α*, where *α* determines whether two variables are independent or not and adjacent variables denote variables connected to the two target variables in PCACMI at the previous step. PCACMI repeats the above steps until there is no higher order connection. The MATLAB code for PCACMI is provided by [21] at the author’s website https://sites.google.com/site/xiujunzhangcsb/software/pca-cmi.

#### Conditional mutual inclusive information-based network inference (CMI2NI)

Recently, Zhang et al. [22] proposed the conditional mutual inclusive information-based network inference (CMI2NI) method that improves the PCACMI method [21]. CMI2NI considers the Kullback-Leibler divergences from the joint probability density function (PDF) of target variables to the interventional PDFs removing the dependency between two variables of interest. Instead of using CMI, CMI2NI uses the conditional mutual inclusive information (CMI2) as the measure of dependency between two variables of interest given other variables. To be specific, we consider three random variables *X*, *Y* and *Z*. For these three random variables, the CMI2 between *X* and *Y* given *Z* is defined as

9 $$  \text{CMI2}(X,Y|Z) = \left(D_{\text{KL}}(P || P_{X \rightarrow Y}) + D_{\text{KL}}(P || P_{Y \rightarrow X}) \right)/2,  $$

where *D*_KL_(*f*||*g*) is the Kullback-Leibler divergence from *f* to *g*, *P* is the joint PDF of *X*, *Y* and *Z*, and *P*_*X*→*Y*_ is the interventional probability of *X*, *Y* and *Z* for removing the connection from *X* to *Y*.

With Gaussian assumption on the observed data, the CMI2 for two random variables *X* and *Y* given *m*-dimensional vector *Z* can be expressed as

10 $$ \begin{aligned} CMI2(X,Y|Z) &= \frac{1}{4}\left(\text{tr}(C^{-1} \Sigma) + \text{tr}(\tilde{C}^{-1} \tilde{\Sigma}) + \log C_{0}\right.\\ &\left.\qquad+ \log \tilde{C}_{0} - 2n \right), \end{aligned}  $$

where *Σ* is the covariance matrix of (*X*,*Y*,*Z* ^*T*^)^*T*^, $\tilde {\Sigma }$ is the covariance matrix of (*Y*,*X*,*Z* ^*T*^)^*T*^, *Σ*_*XZ*_ is the covariance matrix of (*X*,*Z* ^*T*^)^*T*^, *Σ*_*YZ*_ is the covariance matrix of (*Y*,*Z* ^*T*^)^*T*^, *n*=*m*+2, and *C*, $\tilde {C},C_{0}$ and $\tilde {C}_{0}$ are defined with the elements of $\Sigma,\Sigma _{XZ},\Sigma _{YZ},\Sigma ^{-1},\Sigma _{XZ}^{-1}$ and $\Sigma _{YZ}^{-1}$ (see Theorem 1 in [22] for details). As applied in PCACMI, CMI2NI adopts the path consistency algorithm (PCA) to efficiently calculate the CMI2 estimates. All steps of the PCA in CMI2NI are the same as one of PCACMI if we change the CMI to the CMI2. In the PCA steps of CMI2NI, two variables are regarded as independent if the corresponding CMI2 estimate is less than a given threshold *α*. The MATLAB code for CMI2NI is available at the author’s website https://sites.google.com/site/xiujunzhangcsb/software/cmi2ni.

#### Sparse partial correlation estimation (SPACE)

In the Gaussian graphical models [45], the conditional dependencies among *p* variables can be represented by a graph $\mathcal {G}=\left (V,E\right)$, where *V*={1,2,…,*p*} is a set of nodes representing *p* variables and *E*={(*i*,*j*) | *ω*_*ij*_≠0, 1≤*i*≠*j*≤*p*} is a set of edges corresponding to the nonzero off-diagonal elements of *Ω*.

To describe the SPACE method, we consider linear models such that for *i*=1,2,…,*p*,

11 $$ \mathbf{X}_{i}=\sum\limits_{j\neq i}\beta_{ij}\mathbf{X}_{j}+\boldsymbol{\epsilon}_{i}  $$

where ***ε***_*i*_ is an *n*-dimensional random vector from the multivariate normal distribution with mean **0** and covariance matrix (1/*ω*_*ii*_)*I*_*n*_, and *I*_*n*_ is an identity matrix with size of *n*×*n*. Under normality, the regression coefficients *β*_*ij*_s can be replaced with the partial correlations *ρ* ^*ij*^s by the relationship

12 $$ \beta_{ij}=-\frac{\omega_{ij}}{\omega_{ii}}=\rho^{ij}\sqrt{\frac{\omega_{jj}}{\omega_{ii}}},  $$

where $\rho ^{ij}=\text {corr}\left (X_{i},X_{j}~|~X_{k},k\neq i,j\right)=-{\omega _{ij}}\left /{\sqrt {\omega _{ii}\omega _{jj}}}\right.$ is a partial correlation between *X*_*i*_ and *X*_*j*_. Motivated by the relationship (12), Peng et al. [18] propose the SPACE method for solving the following *ℓ*_1_-regularized problem:

13 $$ {\begin{aligned} \min_{\rho}\frac{1}{2}\sum\limits_{i=1}^{p}\left\{ w_{i}\sum\limits_{k=1}^{n}\left(X_{i}^{k}-\sum\limits_{j\neq i}\rho^{ij}\sqrt{\frac{\omega_{jj}}{\omega_{ii}}}X_{j}^{k}\right)^{2}\right\} +\lambda\sum\limits_{1\le i<j \le p}|\rho^{ij}|, \end{aligned}}   $$

where *w*_*i*_ is a nonnegative weight for the *i*-th squared error loss.

### Proposed approach incorporating previously known hub information

#### Extended sparse partial correlation estimation (ESPACE)

**Fig. 1:**
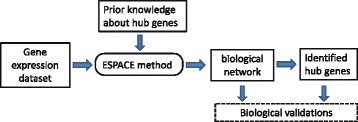
Flowchart of ESPACE

In this paper, we assume that some genes (or nodes), which are referred to as hub genes (or hub nodes), regulate many other genes, and we also assume that many of these hub genes were identified from previous experiments. To incorporate information about hub nodes, we propose the extended SPACE (ESPACE) method, which extends the model space by using an additional tuning parameter *α* on edges connected to the given hub nodes. This additional tuning parameter can reflect the hub gene information by reducing the penalty on edges connected to hub nodes. To be specific, let $\mathcal {H}$ be the set of hub nodes that were previously identified. The ESPACE method we propose solves

14 $$ {\begin{aligned} &\min_{\rho}\frac{1}{2}\sum\limits_{i=1}^{p}\left\{w_{i} \sum\limits_{k=1}^{n}\left(X_{i}^{k}-\sum\limits_{j\neq i}\rho^{ij}\sqrt{\frac{\omega_{jj}}{\omega_{ii}}}X_{j}^{k}\right)^{2}\right\}\\ &\quad+\alpha \lambda \sum\limits_{{i<j,\atop \{i \in \mathcal{H}\} \cup \{j\in \mathcal{H}\}}}|\rho^{ij}|+ \lambda \sum\limits_{ {i<j,\atop i,j\in \mathcal{H}^{c}}}|\rho^{ij}|, \end{aligned}}   $$

where 0<*α*≤1. Note that we consider the weights *w*_*i*_s for the squared error loss as one in this paper. To summarize the process of the proposed method, we depict the flowchart of the ESPACE method in Fig. 1. As described in Fig. 1, the ESPACE has the prior knowledge about hub genes as an additional input, which is the novelty of the proposed method compared to the other existing methods.

#### Extended graphical lasso (EGLASSO)

In the Background, we mentioned the proposed procedure is applicable to other methods such as the graphical lasso. For the purpose of fair comparison and the investigation of the performance, we also applied the proposed strategy to the GLASSO, which is the GLASSO incorporating the hub gene information. We call this procedure the extended graphical lasso (EGLASSO). Similar to the ESPACE, the EGLASSO maximizes

15 $$  \log |\Omega| - \text{tr}(S\Omega) -\alpha \lambda \sum\limits_{{i<j,\atop \{i \in \mathcal{H}\} \cup \{j\in \mathcal{H}\}}}|\omega_{ij}| - \lambda \sum\limits_{{i<j,\atop i,j\in \mathcal{H}^{c}}}|\omega_{ij}|,  $$

where *λ*≥0 and 0<*α*≤1 are two tuning parameters, *S* is the sample covariance matrix, tr(*A*) is the trace of *A* and $\mathcal {H}$ is the set of hub nodes that were previously identified. Note that we can use the R package glasso version 1.7 for the EGLASSO by defining the penalty matrix corresponding to the penalty term in (15).

### Active shooting algorithm for ESPACE

To solve (14), we adopt the active shooting algorithm introduced in [18]. We rewrite the problem (14) as

16 $$  \min_{\rho}\frac{1}{2}\left\|\mathbf{Y}-\tilde{\mathbf{X}}\boldsymbol{\rho}\right\|_{2}^{2}+\alpha \lambda \sum\limits_{{i<j,\atop \{i \in \mathcal{H}\} \cup \{j\in \mathcal{H}\}}}|\rho^{ij}|+ \lambda \sum\limits_{ {i<j,\atop i,j\in \mathcal{H}^{c}}}|\rho^{ij}|,   $$

where $\mathbf {Y}=(\mathbf {X}_{1}^{T},\mathbf {X}_{2}^{T},\ldots,\mathbf {X}_{p}^{T})^{T}$ is an *n*×*p* column vector; $\mathbf {X}^{k,l}=\left (\mathbf {0}_{n(k-1)\times 1}^{T},\mathbf {X}_{l(k)}^{T},\mathbf {0}_{n(l-k-1)\times 1}^{T},\mathbf {X}_{k(l)}^{T},\mathbf {0}_{n(p-l)\times 1}^{T}\right)^{T}$ is an *n*×*p* column vector as well, with $\mathbf {X}_{k(l)}=\sqrt {\frac {\omega _{kk}}{\omega _{ll}}}\mathbf {X}_{k};$

$$ \tilde{\mathbf{X}}= \left(\begin{array}{cccccccc} \mathbf{X}^{1,2}; & \mathbf{X}^{1,3}; & \cdots; & \mathbf{X}^{1,p}; & \mathbf{X}^{2,3}; & \mathbf{X}^{2,4}; & \cdots; & \mathbf{X}^{(p-1),p} \end{array}\right), $$

and ***ρ***=(*ρ* ^12^;*ρ* ^13^;…;*ρ* ^1*p*^;*ρ* ^23^;*ρ* ^24^;…;*ρ* ^(*p*−1)*p*^)^*T*^. Let $\widehat {\boldsymbol {\rho }}^{(m)}$ and $\widehat {\omega }_{ii}^{(m)}$ be estimates of *ρ* and *ω*_*ii*_ at the *m*-th iteration, respectively. Then, the steps of the modified algorithm are outlined below:

- Step 1: (Initialization of $\widehat {\omega }_{ii}$) For *i*=1,2,…,*p*, $\widehat {\omega }_{ii}^{(0)} = 1$ and *s*=0.
- Step 2: (Initialization of $\widehat {\boldsymbol {\rho }}$) For 1≤*i*<*j*≤*p* and *m*=0,
  $$ \begin{array}{llll} \widehat{\rho}^{ij,(0)} &=& \text{sign}\left(\mathbf{Y}^{T}\mathbf{X}^{i,j}\right)\frac{\left(\left|\mathbf{Y}^{T}\mathbf{X}^{i,j}\right|-\alpha\lambda\right)_{+}}{\left(\mathbf{X}^{i,j}\right)^{T}\mathbf{X}^{i,j}} &\text{for}~\{i \in \mathcal{H}\}\cup\{ j \in \mathcal{H}\},\\ \widehat{\rho}^{ij,(0)} &=& \text{sign}\left(\mathbf{Y}^{T}\mathbf{X}^{i,j}\right)\frac{\left(\left|\mathbf{Y}^{T}\mathbf{X}^{i,j}\right|-\lambda\right)_{+}}{\left(\mathbf{X}^{i,j}\right)^{T}\mathbf{X}^{i,j}} &\text{for}~i,j\in\mathcal{H}^{c}, \end{array} $$
  where (*x*)_+_= max(*x*,0) and **X** ^*i*,*j*^s are defined in (16) with $\widehat {\omega }_{ii}^{(s)}$.
- Step 3: Define an active set $\Lambda =\{(i,j)~|~\widehat {\rho }^{ij,(m)}\neq 0\}$.
- Step 4: Iteratively update $\widehat {\boldsymbol {\rho }}^{(m)}$ for (*k*,*l*)∈*Λ*,
  $$ \begin{array}{lll} \widehat{\rho}^{kl,(m)}&= \text{sign}\left((\mathbf{X}^{k,l})^{T}\boldsymbol{\epsilon}'\right)\frac{\left(\left|(\mathbf{X}^{k,l})^{T}\boldsymbol{\epsilon}'\right|-\alpha\lambda\right)_{+}}{(\mathbf{X}^{k,l})^{T}\mathbf{X}^{k,l}}\\ &\quad\text{for}~\{k \in \mathcal{H}\}\cup\{ l \in \mathcal{H}\}, \\ \medskip \widehat{\rho}^{kl,(m)}&= \text{sign}\left((\mathbf{X}^{k,l})^{T}\boldsymbol{\epsilon}'\right)\frac{\left(\left|(\mathbf{X}^{k,l})^{T}\boldsymbol{\epsilon}'\right|-\lambda\right)_{+}}{(\mathbf{X}^{k,l})^{T}\mathbf{X}^{k,l}}&\text{for}~k,l\in\mathcal{H}^{c}, \end{array} $$
  where $\boldsymbol {\epsilon }'= \mathbf {Y}-{\sum \nolimits }_{(i,j)\neq (k,l)} \tilde {\rho }^{ij}\mathbf {X}^{i,j}$ and $\tilde {\rho }^{ij}$s are current estimates at the step for updating the (*k*,*l*)-th partial correlation.
- Step 5: Repeat Step 4 until convergence occurs on the active set *Λ*.
- Step 6: Update $\widehat {\boldsymbol {\rho }}^{(m+1)}$ for 1≤*i*<*j*≤*p* by using the equations in Step 4. If the maximum difference between $\widehat {\boldsymbol {\rho }}^{(m+1)}$ and $\widehat {\boldsymbol {\rho }}^{(m)}$ is less than a pre-determined tolerance *τ*, then go to Step 7 with the estimates $\widehat {\boldsymbol {\rho }}^{(m+1)}$. Otherwise, consider *m*=*m*+1 and go back to Step 3.
- Step 7: Update $\widehat {\omega }_{ii}^{(s+1)}$ for *i*=1,2,…,*p*,
  $$\begin{aligned} \frac{1}{\widehat{\omega}_{ii}^{(s+1)}}&= \frac{1}{n}\left\|\mathbf{X}_{i}-\sum\limits_{j\neq i}\widehat{\rho}^{ij,(m+1)}\sqrt{\frac{\widehat{\omega}_{jj}^{(s)}}{\widehat{\omega}_{ii}^{(s)}}}\mathbf{X}_{j}\right\|_{2}^{2}\\ &\quad\text{for}~i=1,2,\ldots,p. \end{aligned} $$
- Step 8: Repeat Step 2 through Step 7 with *s*=*s*+1 until convergence occurs on $\widehat {\omega }_{ii}$s.

Note that the number of iterations of $\widehat {\omega }_{ii}$s is usually small for stabilization of the estimates of *ρ*. In our numerical study, the estimates of *ω*_*ii*_s converge within 10 iterations. Moreover, the inner products such as **Y** ^*T*^ **X** ^*i*,*j*^, whose complexity is *O*(*n* *p*), can efficiently be computed by rewriting $\mathbf {Y}^{T}\mathbf {X}^{i,j} = {\sum \nolimits }_{k=1}^{n} \left (\sqrt {\omega _{jj}/\omega _{ii}} + \sqrt {\omega _{jj}/\omega _{ii}}\right) X_{i}^{k} X_{j}^{k}, $ whose complexity is *O*(*n*). We implemented the R package espace, which is available from https://sites.google.com/site/dhyeonyu/software.

### Choice of tuning parameters

We have introduced the ESPACE method, which relaxes the penalty on edges connected to the hub genes (i.e., *α*<1) but uses the same penalty on edges connected to non-hub gene (i.e., *α*=1). When no hub genes are involved in a network, ESPACE is reduced to SPACE. For a given *λ*, this modification allows us to find more edges connected to the hub genes by reducing *α*. In practice, however, we do not know the values of *λ* and *α*. In this paper, we consider the GIC-type criterion used in [46] for the Gaussian graphical model to choose the optimal tuning parameters (*λ* ^∗^,*α* ^∗^). Let $\widehat {\rho }_{(\lambda,\alpha)}^{ij}$ be the (*i*,*j*)-th estimate of partial correlation for given *λ* and *α*. The GIC-type criterion is defined as

$${\begin{aligned} \text{GIC}(\lambda,\alpha)&=\sum\limits_{i=1}^{p}\left\{ n\cdot\log {RSS}_{i}+\log{\log{n}}\log (p-1){\vphantom{\widehat{\rho}_{\lambda,\alpha}^{ij}}}\right.\\&\qquad\quad\times\left.\left|\left\{j:j\neq i,\widehat{\rho}_{\lambda,\alpha}^{ij}\neq0\right\}\right|\right\}, \end{aligned}} $$

where ${RSS}_{i} = \left \|\mathbf {X}_{i}-\sum \limits _{j\neq i}\widehat {\rho }_{(\lambda,\alpha)}^{ij}\mathbf {X}_{j(i)}\right \|_{2}^{2}$ and |*A*| denotes a cardinality of a set *A*. We choose the tuning parameters which minimize the GIC-type criterion,

$$(\lambda^{*},\alpha^{*})={\operatornamewithlimits{argmin}_{\lambda,\alpha}\text{GIC}(\lambda,\alpha).} $$

### Simulation studies

#### Simulation settings

**Fig. 2:**
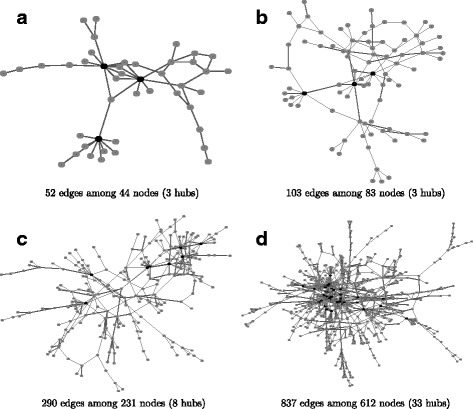
The network structures of the four simulated networks. The structure of the real protein-protein interaction networks [47] were used to construct networks of different sizes by varying the number of references required to support each connection. In the degree distribution, the 0.95 quantile is 7 (connections), so the nodes with more than 7 connections were defined as hub nodes, which are represented as *black nodes* in the network structure. **a** 52 edges among 44 nodes (3 hubs), **b** 103 edges among 83 nodes (3 hubs), **c** 290 edges among 231 nodes (8 hubs) and **d** 837 edges among 612 nodes (33 hubs)
- Step G2: For positive definiteness and symmetry of the concentration matrix, we define a concentration matrix *Ω*=(*ω*_*ij*_)_1≤*i*,*j*≤*p*_ as
  $$\Omega=\frac{1}{2}\left(A+A^{T}\right), $$
  where *A*=(*a*_*ij*_)_1≤*i*,*j*≤*p*_, $a_{ij}=\tilde {\omega }_{ij}/(1.5\cdot d_{i})$ and $d_{i}=\sum \limits _{k\neq i}|\tilde {\omega }_{ik}|$ for *i*=1,2,…,*p*.
- Step G3: Set *ω*_*ii*_=1 for *i*=1,2,…,*p* and *ω*_*ij*_ = 0.1·sign(*ω*_*ij*_) if 0<|*ω*_*ij*_|<0.1.

In this simulation, we consider four real protein-protein interaction (PPI) networks used in a comparative study [24], which were partially selected from the human protein reference database [47]. As mentioned earlier, genes whose degrees are greater than 7 and above the 0.95 quantile of the degree distribution are thought of as hub genes. Figure 2 shows the four PPI networks and their hub genes. Let *p* be the number of nodes in a network. We consider the number of samples as *p*/2 and *p* and generate samples from the multivariate normal distribution with mean **0** and covariance matrix *Σ* defined with $(\Sigma)_{ij} = (\Omega ^{-1})_{ij}/\sqrt {(\Omega ^{-1})_{ii}(\Omega ^{-1})_{jj}}$, where *Ω* is a concentration matrix corresponding to a given network structure. To generate a positive definite concentration matrix, we use the following procedure as described in [18]:

- Step G1: For a given edge set *E*, we generate an initial concentration matrix $\tilde {\Omega }=(\tilde {\omega }_{ij})_{1\le i,j\le p}$ with
  $$\tilde{\omega}_{ij}=\left\{ \begin{array}{ll} 1 & \quad i=j\\ 0 & \quad i\neq j,~(i,j)\notin E\\ \sim~Unif(D) & \quad i\neq j,~(i,j)\in E \end{array}\right., $$
  where *D*=[−1,−0.5]∪[ 0.5,1].

With these four networks, we have conducted the numerical comparisons of the ESPACE and the SPACE methods, as well as seven other methods including the other reviewed existing methods and EGLASSO. For the purpose of fair comparison, we select the optimal model by the GIC for SPACE, ESPACE, GLASSO, GLASSO-SF, and EGLASSO. Since there is no specific rule for the model selection in the other methods, we set the level *α*=0.2 for GeneNet and NS, and the threshold *α*=0.03 for PCACMI and CMI2NI. Note that the pre-determined level *α*=0.2 is a default of the GeneNet package and used in [35]. The pre-determined threshold *α*=0.03 was used in [21, 22].

Note that all the existing methods need *O*(*p* ^2^) memory space to store and calculate values corresponding to the interactions between variables. We can reduce this memory consumption when the whole variables can be divided into several conditionally independent blocks by using the condition described in [16].

#### Sensitivity analysis on random noise in the observed data

To investigate the effect of the random noise contained in the observed data, we consider sensitivity analysis for the variance of the random noise. To be specific, suppose that a random vector **X**=(*X*_1_,*X*_2_,…,*X*_*p*_)^*T*^ follows the multivariate normal distribution with mean **0** and covariance matrix *Σ*, a vector of random noise *ε*=(*ε*_1_,*ε*_2_,…,*ε*_*p*_)^*T*^ follows the multivariate normal distribution with mean **0** and covariance matrix $\sigma _{\epsilon }^{2} I$, and **X** and *ε* are independent, where *I* is the identity matrix. Furthermore, we assume that an observed random vector **Z**=(*Z*_1_,*Z*_2_,…,*Z*_*p*_)^*T*^ such that

17 $$ \mathbf{Z} = \mathbf{X} + \varepsilon.  $$

Thus, the covariance matrix of **Z** becomes $\Sigma + \sigma _{\epsilon }^{2} I$, which may have a different conditional dependent structure to one of **X**.

For example, if we consider $\sigma _{\epsilon }^{2} = 0.5$ and the following *Σ* and *Σ*_**Z**_

18 $$ \Sigma= \left(\begin{array}{ccc} 15/11 & -8/11 & 2/11 \\ -8/11& 16/11& -4/11 \\ 2/11& -4/11& 12/11 \\ \end{array}\right),~ \Sigma_{\mathbf{Z}}= \Sigma + \sigma_{\epsilon}^{2} I,  $$

then the inverse matrices of *Σ* and *Σ*_**Z**_ are calculated as

19 $$ {\begin{aligned} &\Sigma^{-1}= \left(\begin{array}{ccc} 1 & 0.5 & 0 \\ -0.5& 1& 0.25 \\ 0& 0.25& 1 \\ \end{array}\right)\text{and}\\ &\Sigma_{\mathbf{Z}}^{-1}= \left(\begin{array}{ccc} 0.63 &0.23 &-0.02 \\ 0.23 &0.62 & 0.12 \\ -0.02& 0.12 & 0.66 \end{array}\right), \text{respectively.} \end{aligned}}  $$

Thus, we can see that *Z*_1_ and *Z*_3_ are conditionally dependent given *Z*_2_ while *X*_1_ and *X*_3_ are conditionally independent given *X*_2_. Moreover, the nonzero partial correlations decrease when the variance of the random noises increases. From these observations, the performance of the estimation becomes worse if the variance of the random noise increases.

In this sensitivity analysis, we consider $\sigma _{\epsilon }^{2} = 0, 0.01, 0.1, 0.25, 0.5$ and *p*=231 and *n*=115,231 with the same network structure as the one of *p*=231 in Fig. 2. To focus on the proposed method, we apply the SPACE and the ESPACE methods to the 50 generated datasets containing random noise having variance $\sigma _{\epsilon }^{2}$.

#### Performance measures

To investigate the gains from the extension, we use five performance measures: sensitivity (SEN), specificity (SPE), false discovery rate (FDR), mis-specification rate (MISR) and Matthews correlation coefficients (MCC). Note that the MCC, which lies between −1 and +1, has been used to measure the performance of binary classification, where +1, 0, and −1 denote a perfect classification, a random classification, and a total discordance of classification, respectively. Let *ρ* and $\widehat {\rho }_{\lambda,\alpha }$ be (*p*(*p*−1)/2)-dimensional vectors of the true and estimated partial correlation, respectively. The above five measures are defined as

$${\begin{aligned} \begin{array}{l} \text{SEN} \equiv \text{TP}/(\text{TP}+\text{FN}),~~ \text{SPE} \equiv \text{TN}/(\text{TN}+\text{FP}), \\ \text{FDR} \equiv \text{FP}/(\text{TP} + \text{FP}),~~ \text{MISR} \equiv (\text{FN} + \text{FP})/\left(p(p-1)/2\right)~~\text{and}\\ \text{MCC} \equiv \frac{\text{TP} \times \text{TN} - \text{FP} \times \text{FN}}{\sqrt{(\text{TP}+\text{FP})(\text{TP}+\text{FN})(\text{TN}+\text{FP})(\text{TN}+\text{FN})}}, \end{array} \end{aligned}} $$

where $\text {TP} = {\sum \nolimits }_{i<j} I(\rho ^{ij} \neq 0) I(\widehat {\rho }^{ij}_{\lambda,\alpha } \neq 0)$, $\text {FP} = {\sum \nolimits }_{i<j} I(\rho ^{ij} = 0) I(\widehat {\rho }^{ij}_{\lambda,\alpha } \neq 0)$, $\text {FN} = {\sum \nolimits }_{i<j} I(\rho ^{ij} \neq 0) I(\widehat {\rho }^{ij}_{\lambda,\alpha }=0)$ and $\text {TN} = {\sum \nolimits }_{i<j} I(\rho ^{ij} = 0) I(\widehat {\rho }^{ij}_{\lambda,\alpha } = 0)$.

### Application to *Escherichia coli* dataset

We applied the ESPACE method to the largest public *Escherichia coli* (*E.coli*) microarray dataset available from the Many Microbe Microarrays database (M3D) [48]. The M3D contains 907 microarrays measured under 466 experimental conditions using Affymetrix GeneChip *E.coli* genome arrays. Microarrays from the same experimental conditions were averaged to derive the mean expression. The data set (“E_coli_v4_Build_6” from the M3D) contains the expression levels of 4297 genes from 446 samples. In the *E.coli* genome, a number of studies have been conducted to identify transcriptional regulations. The RegulonDB [49] curates the largest and best-known information on the transcriptional regulation of *E.coli*. To combine the information from the above two databases, we focus on the 1623 genes reported in both the M3D and the RegulonDB. As mentioned before, the TFs are known to regulate many other genes in the genome and can be considered potential hubs. To incorporate the information about the potential hubs, we used a list of 180 known TF-encoding genes from the RegulonDB. The RegulonDB also provides 3811 transcriptional interactions among the 1623 genes, which were used as the gold standard to evaluate the accuracy of the constructed networks.

### Application to lung cancer adenocarcinoma dataset

Lung cancer is the leading cause of death from cancer, both in the United States and worldwide; it has a 5-year survival rate of approximately 15% [50]. The progression and metastasis of lung cancer varies greatly among early stage lung cancer patients. To customize treatment plans for individual patients, it is important to identify prognostic or predictive biomarkers, which allows for more precise classification of lung cancer patients. In this study, we applied the extended SPACE method to reconstruct the gene regulatory network in lung cancer. Exploring network structures can facilitate comprehension of biological mechanisms underlying lung cancer and identification of important genes that could be potential lung cancer biomarkers. We constructed the gene network using microarray data from 442 lung cancer adenocarcinoma patients in the Lung Cancer Consortium study [51]. For detail about preprocessing this dataset, please refer to [7]. First, univariate Cox regression was used to identify the genes whose expression levels are correlated with patient survival outcomes, after adjusting for clinical factors such as study site, age, gender, and stage. The false discovery rate (FDR) was then calculated using a Beta-Uniform model [52]. By controlling the FDR to less than 10%, we identified 794 genes that were associated with the survival outcome of lung cancer patients. Among these 794 genes, 22 were found to appear among the 236 carefully curated cancer genes of the FoundationOne^TM^ gene panel (Foundation Medicine, Inc.). Current biological knowledge indicates genes from this panel play a key role in various types of cancer. These 22 genes were then input as known hub genes to the ESPACE method.

## Results and discussion

### Simulation results

#### Comparison results for existing methods

For each network, we generated 50 datasets and reconstructed the network from each dataset using nine different network construction methods, including both the SPACE and the ESPACE methods. In addition to the five performance measures, we also measure the computation time (Time) of each method to compare the efficiency. Note that all methods are executed on R software [53] for the purpose of fair comparison. We implemented the R codes for PCACMI and CMI2NI using the authors’ MATLAB codes. The computation times are measured in CPU time (seconds) by using a desktop PC (Intel Core(TM) i7-4790K CPU (4.00 GHz) and 32 GB RAM).

Tables 1, 2, 3 and 4 report the averages and standard errors of the number of the estimated edges, the five performance measures of the estimation of the network structures and computation times with the optimal tuning parameter *λ* ^∗^ for SPACE, GLASSO, GLASSO-SF; the optimal tuning parameters *α* ^∗^ and *λ* ^∗^ for ESPACE and EGLASSO; and the pre-determined *α* for GeneNet, NS, PCACMI, and CMI2NI.

**Table 1:** The averages of the number of estimated edges, the five performance measures and the computation time (sec.) over 50 datasets

*p*	*n*	Method	$|\hat {E}|$	SEN	SPE	FDR	MISR	MCC	Time
44	22	GeneNet	0.80	1.00	99.97	4.38	5.47	3.25	0.02
(|*E*|=52)			(0.31)	(0.37)	(0.02)	(2.05)	(0.01)	(1.06)	(0.00)
		NS	0.66	1.19	100.00	2.50	5.44	6.65	0.03
			(0.13)	(0.23)	(0.00)	(2.05)	(0.01)	(1.16)	(0.00)
		SPACE	10.48	12.50	99.55	33.38	5.23	24.58	0.01
			(1.21)	(1.32)	(0.07)	(3.40)	(0.05)	(1.69)	(0.00)
		ESPACE	12.06	16.50	99.61	23.62	4.96	31.46	0.00
			(1.12)	(1.33)	(0.06)	(2.52)	(0.05)	(1.67)	(0.00)
		GLASSO	6.64	6.88	99.66	33.86	5.44	17.98	0.00
			(0.95)	(0.66)	(0.08)	(4.12)	(0.06)	(1.19)	(0.00)
		GLASSO-SF	7.14	6.77	99.60	32.51	5.51	17.39	0.04
			(1.20)	(0.70)	(0.10)	(4.11)	(0.07)	(1.12)	(0.00)
		EGLASSO	6.34	8.65	99.79	26.36	5.22	22.14	0.00
			(0.74)	(0.96)	(0.04)	(3.47)	(0.04)	(1.49)	(0.00)
		PCACMI	55.60	33.15	95.71	68.93	7.73	27.98	0.21
			(0.62)	(0.73)	(0.07)	(0.65)	(0.08)	(0.71)	(0.01)
		CMI2NI	59.34	35.38	95.42	68.92	7.88	28.97	0.50
			(0.73)	(0.70)	(0.07)	(0.57)	(0.08)	(0.63)	(0.07)
	44	GeneNet	9.28	13.58	99.75	12.19	4.99	29.73	0.02
			(1.32)	(1.48)	(0.07)	(2.11)	(0.05)	(1.61)	(0.00)
		NS	4.16	7.65	99.98	3.48	5.10	25.92	0.03
			(0.23)	(0.42)	(0.01)	(1.26)	(0.02)	(0.75)	(0.00)
		SPACE	27.28	37.38	99.12	25.79	4.27	48.78	0.01
			(1.50)	(1.73)	(0.08)	(1.47)	(0.07)	(1.71)	(0.00)
		ESPACE	23.12	34.38	99.41	20.03	4.16	50.01	0.00
			(1.20)	(1.36)	(0.07)	(1.47)	(0.06)	(0.94)	(0.00)
		GLASSO	11.32	13.12	99.50	26.48	5.25	27.20	0.00
			(1.35)	(1.08)	(0.10)	(3.03)	(0.05)	(0.99)	(0.00)
		GLASSO-SF	13.62	14.38	99.31	30.92	5.36	26.77	0.04
			(1.52)	(1.23)	(0.11)	(3.22)	(0.07)	(1.16)	(0.00)
		EGLASSO	14.34	22.38	99.70	16.31	4.55	39.94	0.00
			(1.10)	(1.63)	(0.04)	(1.81)	(0.07)	(1.73)	(0.00)
		PCACMI	26.70	34.00	98.99	33.39	4.58	45.47	0.18
			(0.44)	(0.70)	(0.05)	(1.28)	(0.07)	(0.89)	(0.00)
		CMI2NI	28.84	35.50	98.84	35.75	4.64	45.54	0.27
			(0.48)	(0.67)	(0.04)	(0.96)	(0.06)	(0.74)	(0.01)

The reported values for the SEN, SPE, FDR, MISR and MCC were multiplied by 100. Numbers in the parentheses denote the standard errors

**Table 2:** The averages of the number of estimated edges, the five performance measures and the computation time (sec.) over 50 datasets

*p*	*n*	Method	$|\hat {E}|$	SEN	SPE	FDR	MISR	MCC	Time
83	41	GeneNet	6.04	5.32	99.98	6.57	2.88	19.21	0.05
(|*E*|=103)			(0.61)	(0.52)	(0.00)	(1.64)	(0.01)	(1.46)	(0.00)
		NS	2.36	2.21	100.00	2.83	2.96	13.29	0.09
			(0.20)	(0.19)	(0.00)	(1.42)	(0.01)	(0.82)	(0.01)
		SPACE	4.62	4.04	99.99	6.41	2.92	16.18	0.03
			(0.79)	(0.64)	(0.00)	(2.36)	(0.02)	(1.40)	(0.00)
		ESPACE	7.28	6.37	99.98	5.75	2.86	21.04	0.01
			(0.94)	(0.78)	(0.01)	(1.38)	(0.02)	(1.55)	(0.00)
		GLASSO	11.40	8.78	99.93	16.81	2.83	25.48	0.00
			(0.87)	(0.59)	(0.01)	(2.05)	(0.01)	(0.88)	(0.00)
		GLASSO-SF	9.90	7.53	99.94	16.30	2.86	23.44	0.12
			(0.81)	(0.53)	(0.01)	(2.08)	(0.01)	(0.84)	(0.00)
		EGLASSO	11.28	8.89	99.94	15.36	2.82	25.93	0.00
			(0.86)	(0.59)	(0.01)	(1.86)	(0.01)	(0.88)	(0.00)
		PCACMI	48.44	27.24	99.38	41.86	2.80	38.52	0.54
			(0.82)	(0.55)	(0.02)	(1.00)	(0.03)	(0.68)	(0.01)
		CMI2NI	48.72	27.88	99.39	40.82	2.77	39.35	0.54
			(0.85)	(0.55)	(0.02)	(0.96)	(0.03)	(0.65)	(0.01)
	83	GeneNet	34.74	31.17	99.92	7.05	2.16	52.97	0.06
			(0.83)	(0.61)	(0.01)	(0.74)	(0.02)	(0.50)	(0.00)
		NS	15.14	14.52	99.99	1.07	2.59	37.20	0.15
			(0.43)	(0.41)	(0.00)	(0.38)	(0.01)	(0.55)	(0.01)
		SPACE	51.84	41.03	99.71	16.81	2.07	56.67	0.05
			(1.95)	(1.28)	(0.02)	(1.06)	(0.03)	(1.11)	(0.00)
		ESPACE	52.34	42.45	99.74	15.42	2.00	58.82	0.03
			(1.46)	(0.86)	(0.02)	(0.96)	(0.02)	(0.54)	(0.00)
		GLASSO	27.98	23.24	99.88	12.57	2.44	43.63	0.00
			(1.33)	(0.96)	(0.02)	(1.32)	(0.03)	(0.97)	(0.00)
		GLASSO-SF	28.04	22.82	99.86	14.53	2.47	42.86	0.13
			(1.31)	(0.90)	(0.02)	(1.41)	(0.02)	(0.89)	(0.00)
		EGLASSO	30.06	26.17	99.91	8.91	2.33	47.48	0.00
			(1.33)	(1.03)	(0.01)	(1.15)	(0.03)	(1.01)	(0.00)
		PCACMI	28.44	25.96	99.95	6.09	2.29	48.66	0.56
			(0.44)	(0.49)	(0.01)	(0.79)	(0.02)	(0.61)	(0.00)
		CMI2NI	28.66	26.76	99.97	3.85	2.25	50.02	0.54
			(0.45)	(0.46)	(0.01)	(0.61)	(0.02)	(0.53)	(0.01)

The reported values for the SEN, SPE, FDR, MISR and MCC were multiplied by 100. Numbers in the parentheses denote the standard errors

**Table 3:** The averages of the number of estimated edges, the five performance measures and the computation time (sec.) over 50 datasets

*p*	*n*	Method	$|\hat {E}|$	SEN	SPE	FDR	MISR	MCC	Time
231	115	GeneNet	115.98	38.39	99.98	3.94	0.69	60.46	0.56
(|*E*|=290)			(1.27)	(0.39)	(0.00)	(0.26)	(0.00)	(0.30)	(0.01)
		NS	54.28	18.71	100.00	0.04	0.89	42.99	0.34
			(0.85)	(0.29)	(0.00)	(0.04)	(0.00)	(0.34)	(0.01)
		SPACE	160.38	46.90	99.91	14.78	0.67	62.85	0.36
			(2.39)	(0.43)	(0.01)	(0.69)	(0.00)	(0.28)	(0.01)
		ESPACE	172.74	49.41	99.89	16.95	0.66	63.74	0.13
			(1.58)	(0.39)	(0.00)	(0.41)	(0.00)	(0.31)	(0.00)
		GLASSO	85.78	28.26	99.99	3.87	0.80	51.25	0.03
			(3.64)	(1.12)	(0.00)	(0.41)	(0.01)	(1.03)	(0.00)
		GLASSO-SF	76.64	25.19	99.99	4.03	0.83	48.31	1.06
			(3.26)	(1.01)	(0.00)	(0.46)	(0.01)	(0.98)	(0.02)
		EGLASSO	86.50	28.63	99.99	3.51	0.79	51.71	0.04
			(3.54)	(1.11)	(0.00)	(0.39)	(0.01)	(1.05)	(0.00)
		PCACMI	73.42	25.14	100.00	0.72	0.82	49.70	4.16
			(0.94)	(0.33)	(0.00)	(0.14)	(0.00)	(0.34)	(0.05)
		CMI2NI	73.42	25.17	100.00	0.62	0.82	49.75	6.25
			(0.94)	(0.33)	(0.00)	(0.13)	(0.00)	(0.34)	(0.07)
	231	GeneNet	173.78	56.66	99.96	5.38	0.51	72.99	0.74
			(1.21)	(0.28)	(0.00)	(0.30)	(0.00)	(0.18)	(0.01)
		NS	128.10	44.15	100.00	0.05	0.61	66.22	0.97
			(0.54)	(0.19)	(0.00)	(0.03)	(0.00)	(0.14)	(0.01)
		SPACE	235.54	68.37	99.86	15.62	0.49	75.68	0.60
			(2.20)	(0.35)	(0.01)	(0.50)	(0.00)	(0.22)	(0.00)
		ESPACE	235.86	69.35	99.87	14.55	0.47	76.72	0.23
			(1.99)	(0.32)	(0.01)	(0.49)	(0.00)	(0.23)	(0.01)
		GLASSO	222.38	64.97	99.87	15.00	0.51	74.00	0.07
			(2.62)	(0.47)	(0.01)	(0.54)	(0.00)	(0.20)	(0.00)
		GLASSO-SF	176.66	56.42	99.95	7.12	0.52	72.13	0.70
			(2.11)	(0.35)	(0.01)	(0.52)	(0.00)	(0.24)	(0.02)
		EGLASSO	222.86	65.68	99.88	14.26	0.50	74.74	0.09
			(2.57)	(0.46)	(0.01)	(0.54)	(0.00)	(0.21)	(0.01)
		PCACMI	74.28	25.61	100.00	0.02	0.81	50.36	6.23
			(0.79)	(0.27)	(0.00)	(0.02)	(0.00)	(0.27)	(0.13)
		CMI2NI	74.28	25.61	100.00	0.02	0.81	50.36	7.99
			(0.79)	(0.27)	(0.00)	(0.02)	(0.00)	(0.27)	(0.11)

The reported values for the SEN, SPE, FDR, MISR and MCC were multiplied by 100. Numbers in the parentheses denote the standard errors

**Table 4:** The averages of the number of estimated edges, the five performance measures and the computation time (sec.) over 50 datasets

*p*	*n*	Method	$|\hat {E}|$	SEN	SPE	FDR	MISR	MCC	Time
612	306	GeneNet	597.72	55.42	99.93	22.24	0.27	65.49	3.91
(|*E*|=837)			(4.89)	(0.22)	(0.00)	(0.41)	(0.00)	(0.15)	(0.03)
		NS	343.52	41.00	100.00	0.10	0.26	63.91	5.72
			(0.86)	(0.10)	(0.00)	(0.03)	(0.00)	(0.08)	(0.07)
		SPACE	781.04	66.72	99.88	28.21	0.27	69.02	16.57
			(8.90)	(0.31)	(0.00)	(0.55)	(0.00)	(0.22)	(0.14)
		ESPACE	765.50	67.36	99.89	26.22	0.25	70.35	3.69
			(5.95)	(0.29)	(0.00)	(0.41)	(0.00)	(0.22)	(0.05)
		GLASSO	1097.32	65.66	99.71	49.86	0.45	57.15	4.86
			(6.08)	(0.18)	(0.00)	(0.25)	(0.00)	(0.17)	(0.37)
		GLASSO-SF	1069.56	60.67	99.70	52.38	0.48	53.45	29.36
			(14.93)	(0.64)	(0.01)	(0.29)	(0.00)	(0.27)	(1.85)
		EGLASSO	1042.64	67.86	99.74	45.43	0.40	60.63	7.59
			(8.54)	(0.34)	(0.00)	(0.28)	(0.00)	(0.18)	(0.65)
		PCACMI	272.08	27.40	99.98	15.70	0.35	47.94	42.09
			(0.99)	(0.11)	(0.00)	(0.24)	(0.00)	(0.14)	(1.41)
		CMI2NI	297.72	29.12	99.97	18.12	0.35	48.70	68.89
			(1.19)	(0.11)	(0.00)	(0.22)	(0.00)	(0.13)	(1.71)
	612	GeneNet	727.94	68.80	99.92	20.80	0.22	73.69	5.56
			(4.53)	(0.20)	(0.00)	(0.32)	(0.00)	(0.13)	(0.06)
		NS	453.50	54.14	100.00	0.08	0.21	73.47	25.69
			(1.19)	(0.14)	(0.00)	(0.02)	(0.00)	(0.10)	(0.38)
		SPACE	983.38	84.01	99.85	28.39	0.22	77.43	62.72
			(6.38)	(0.24)	(0.00)	(0.34)	(0.00)	(0.17)	(1.41)
		ESPACE	983.84	84.96	99.85	27.66	0.21	78.28	17.86
			(4.88)	(0.19)	(0.00)	(0.27)	(0.00)	(0.14)	(0.63)
		GLASSO	1467.52	85.61	99.60	51.15	0.47	64.47	29.86
			(5.04)	(0.11)	(0.00)	(0.17)	(0.00)	(0.12)	(1.21)
		GLASSO-SF	1615.16	85.60	99.52	55.61	0.55	61.42	117.78
			(6.56)	(0.13)	(0.00)	(0.17)	(0.00)	(0.13)	(3.68)
		EGLASSO	1385.60	87.87	99.65	46.89	0.40	68.14	38.67
			(5.37)	(0.12)	(0.00)	(0.20)	(0.00)	(0.14)	(1.35)
		PCACMI	273.22	27.68	99.98	15.20	0.35	48.33	39.25
			(0.98)	(0.11)	(0.00)	(0.17)	(0.00)	(0.12)	(0.59)
		CMI2NI	298.38	29.55	99.97	17.10	0.34	49.37	59.62
			(0.97)	(0.09)	(0.00)	(0.11)	(0.00)	(0.09)	(0.84)

The reported values for the SEN, SPE, FDR, MISR and MCC were multiplied by 100. Numbers in the parentheses denote the standard errors

Overall, ESPACE has the best performance in estimating network structures in terms of the MCC and the MISR except for the case (*p*,*n*)=(83,41), where ESPACE has the second smallest FDR while the MCC and the MISR of ESPACE show the moderate performance among all methods. In the case (*p*,*n*)=(83,41), the CMI-based methods have better performance than the others in terms of the MCC and the MISR, but the CMI-based methods also have the large FDRs (≈41*%*) more than double of those of the other methods. As we described in the Methods Section, the MCC has been used to measure the performance of binary classification and the MISR denotes the total error rate. Thus, this comparison results show that ESPACE is favorable for the identification of edges for the networks with high-dimensional data.

In addition, we made several interesting observations from the results of the our simulation study. First, ESPACE and EGLASSO improve SPACE and GLASSO in terms of the FDR, the MISR, and the MCC for almost scenarios, respectively. The only exception is the case (*p*,*n*)=(231,115) for the ESPACE and the SPACE methods. In this case, although the FDR of ESPACE increases 2.17% compared to one of SPACE, ESPACE still improves SPACE in terms of the SEN, the MISR, and the MCC. This suggests that our proposed strategy, which incorporates the previously known hub information, can reduce the errors in estimating network structures compared to the existing method without considering known hub information. Second, GeneNet controls the FDR relatively close to the given level *α* while the FDRs of NS are controlled conservatively. For instance, the FDRs of GeneNet are measured between 3.94 and 22.24% and NS has the FDRs less than 3.48%. Note that GeneNet and NS control the FDR under 20% (*α*=0.2) in this simulation study. Third, all methods except the CMI-based methods (the PCACMI and the CMI2NI) have similar efficiency for the relatively low dimensions (*p*=44,83). The CMI-based methods are relatively slower than the other methods for all the scenarios except for the case (*p*,*n*)=(612,612), where GLASSO-SF is the slowest and 1.4 times slower than CMI2NI. CMI2NI is slightly slower than PCACMI for the relatively high dimensions (*p*=231,612). Finally, even though ESPACE is not the fastest method among the nine methods we consider, there is no overall winner and ESPACE is the third best in terms of the computation time for *p*=231,612 except for the case (*p*,*n*)=(612,612) where ESPACE is faster than SPACE, GLASSO-SF, PCACMI and CMI2NI.

**Fig. 3:**
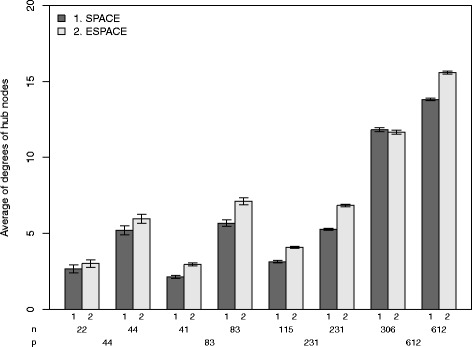
Plots of the averages of degrees of hub nodes over the simulated 50 datasets. *Vertical lines* denote 95% confidence intervals of the averages

To investigate the other property of the proposed approach, we depict barplots of the averages of degrees of known hub genes over 50 datasets for ESPACE and SPACE in Fig. 3. Figure 3 shows that ESPACE tends to find more edges connected to known hub genes than SPACE. The only exception is the case (*p*,*n*)=(612,306), where the average by the ESPACE is 0.57 less than that by SPACE. We conjecture this is simply due the difference in the number of estimated edges, which by ESPACE is 15.54 less than that of SPACE on average. This property is due to the result that the averages of *α* ^∗^ selected by the GIC in the ESPACE method lie between 0.76 and 0.97 for all the scenarios, which indicates that ESPACE has incorporated prior information about the hub genes and reduced the penalty on edges connected to known hub genes.

#### Results of sensitivity analysis on random noise

Table 5 reports the averages of the number of the estimated edges and the five performance measures. From the results in Table 5, we can see that the performance of estimation decreases when the variance of the random noises increases in both the SPACE and the ESPACE. For a relatively small sample size (*n*=115), both the SPACE and the ESPACE are more sensitive to the variance $\sigma _{\epsilon }^{2}$ compared to the case of *n*=231. Even though the performance of two methods decreases by similar amounts as the variance $\sigma _{\epsilon }^{2}$ increases, the ESPACE has better performance than the SPACE in terms of the MCC and the MISR.

**Table 5:** The averages of the number of estimated edges and the five performance measures over 50 datasets

*p*	*n*	$\sigma _{\epsilon }^{2}$	Method	$|\hat {E}|$	SEN	SPE	FDR	MISR	MCC
231	115	0	SPACE	160.38	46.90	99.91	14.78	0.67	62.85
(|*E*|=290)				(2.39)	(0.43)	(0.01)	(0.69)	(0.00)	(0.28)
			ESPACE	172.74	49.41	99.89	16.95	0.66	63.74
				(1.58)	(0.39)	(0.00)	(0.41)	(0.00)	(0.31)
		0.01	SPACE	156.00	45.97	99.91	14.06	0.68	62.45
				(2.79)	(0.57)	(0.01)	(0.66)	(0.00)	(0.33)
			ESPACE	153.16	45.79	99.92	12.85	0.67	62.78
				(2.64)	(0.55)	(0.01)	(0.67)	(0.00)	(0.34)
		0.1	SPACE	106.08	32.61	99.96	9.15	0.78	53.07
				(5.11)	(1.41)	(0.00)	(0.80)	(0.01)	(1.21)
			ESPACE	104.30	32.31	99.96	8.64	0.78	53.15
				(4.96)	(1.37)	(0.00)	(0.73)	(0.01)	(1.10)
		0.25	SPACE	44.76	14.81	99.99	3.63	0.94	37.27
				(1.75)	(0.54)	(0.00)	(0.42)	(0.01)	(0.62)
			ESPACE	49.14	16.12	99.99	4.59	0.92	38.79
				(1.47)	(0.45)	(0.00)	(0.41)	(0.00)	(0.53)
		0.5	SPACE	55.88	14.97	99.95	22.20	0.98	33.81
				(0.90)	(0.25)	(0.00)	(0.72)	(0.00)	(0.37)
			ESPACE	57.34	15.32	99.95	22.49	0.97	34.14
				(1.03)	(0.31)	(0.00)	(0.69)	(0.00)	(0.44)
	231	0	SPACE	235.54	68.37	99.86	15.62	0.49	75.68
				(2.20)	(0.35)	(0.01)	(0.50)	(0.00)	(0.22)
			ESPACE	235.86	69.35	99.87	14.55	0.47	76.72
				(1.99)	(0.32)	(0.01)	(0.49)	(0.00)	(0.23)
		0.01	SPACE	230.90	67.54	99.87	15.00	0.49	75.50
				(2.11)	(0.34)	(0.01)	(0.46)	(0.00)	(0.21)
			ESPACE	231.86	68.54	99.87	14.05	0.47	76.49
				(2.21)	(0.33)	(0.01)	(0.55)	(0.01)	(0.24)
		0.1	SPACE	214.30	62.28	99.87	15.48	0.54	72.25
				(2.22)	(0.37)	(0.01)	(0.53)	(0.00)	(0.25)
			ESPACE	214.04	63.22	99.88	14.07	0.52	73.41
				(2.33)	(0.35)	(0.01)	(0.56)	(0.00)	(0.22)
		0.25	SPACE	184.76	54.10	99.89	14.77	0.61	67.57
				(2.38)	(0.43)	(0.01)	(0.60)	(0.00)	(0.27)
			ESPACE	181.02	54.52	99.91	12.53	0.58	68.78
				(1.42)	(0.27)	(0.00)	(0.42)	(0.00)	(0.21)
		0.5	SPACE	112.28	35.38	99.96	7.89	0.74	56.55
				(3.23)	(0.82)	(0.00)	(0.61)	(0.01)	(0.54)
			ESPACE	123.06	38.58	99.96	8.48	0.71	58.98
				(2.80)	(0.65)	(0.00)	(0.60)	(0.00)	(0.37)

The reported values for the SEN, SPE, FDR, MISR and MCC were multiplied by 100. Numbers in the parentheses denote the standard errors

### Comparison of the identified GRNs in *Escherichia coli* dataset

**Fig. 4:**
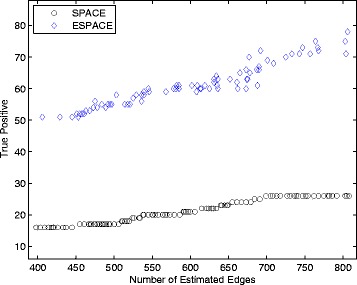
Plot of the number of the TPs vs. the number of the estimated edges for various *λ*s with *α* ^∗^ in Table 6

In this study, we compared the performance of network construction using the SPACE and the ESPACE methods for the model selected by the GIC. We report the number of estimated edges and the true positives, which are matched to the transcriptional interactions in the RegulonDB, in Table 6. The SPACE method estimated 368 edges among 524 genes, which contain 16 TF-encoding genes, and identified 16 transcriptional interactions as true positives. In comparison, the ESPACE method estimated 349 edges among 478 genes containing 29 TF-encoding genes and found 45 transcriptional interactions in the RegulonDB. The ESPACE method found more interactions than the SPACE method and increased the ratio of the number of TPs versus the number of estimated edges as 8.55%. Figure 4 shows the number of TPs vs. the number of estimated edges for various *λ* values with *α* ^∗^ in Table 6. The number of TPs of the ESPACE method is consistently greater than those of the SPACE method at similar sparsity. These results clearly indicate that incorporating potential hub gene information improves the accuracy of network construction.

**Table 6:** Summary of the estimated networks using the SPACE and ESPACE methods from the *E.coli* dataset. We denote a set of estimated edges and a set of the interactions from the RegulonDB by $\widehat {E}$ and T, respectively

Method	*α* ^∗^	*λ* ^∗^	$ |\widehat {E}| $	$~ |\widehat {E} \bigcap \mathrm {T} | ~$	$~ {| \widehat {E} \bigcap \mathrm {T} |}\left / {|\widehat {E}|}\right. ~ $
SPACE	1	806.6	368	16	4.35%
ESPACE	0.85	835.2	349	45	12.89%

### Comparison of the identified GRNs in lung cancer adenocarcinoma dataset

**Fig. 5:**
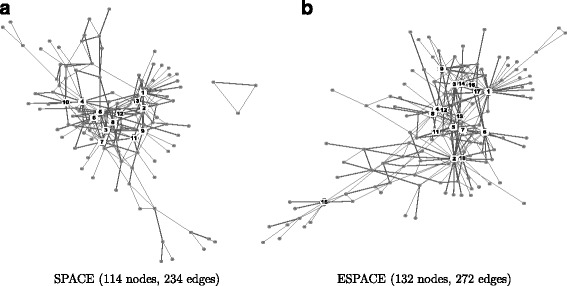
Estimated networks structure using the SPACE and ESPACE methods. The nodes with more than 7 connections (the 0.95 quantile in the degree distribution) were defined as hub nodes, which are represented as black nodes in the network structure. The details of hub genes are reported in Table 7. **a** SPACE (114 nodes, 234 edges) and **b** ESPACE (132 nodes, 272 edges)

We again compared the performances of network construction using the SPACE and the ESPACE methods. An overview of the networks constructed using both methods is shown in Fig. 5. The SPACE method estimated 234 edges between 114 genes and the ESPACE method found 272 edges between 132 genes. Although the numbers of estimated edges from both the SPACE and ESPACE methods are quite similar, 16.7 and 28.3% of the estimated edges in networks by the SPACE and the ESPACE are different, respectively. We identified hub genes using the criterion mentioned at the beginning of this paper. The lists of hub genes identified in both networks are reported in Table 7. Interestingly, all hub genes identified by the SPACE method were also found using ESPACE. Note that this is not the usual case. For instance, if we define a hub as a node whose degree is greater than 5, the set of hub genes identified by the SPACE is not a subset of the hub genes identified by the ESPACE. To investigate the gains of the ESPACE method, therefore, we focused on the hub genes identified only by ESPACE (AURKA, APC, CDKN3), among which, AURKA and APC are among the 22 pre-specidifed hub genes while CDKN3 is not.

**Table 7:** Hub genes from the estimated graphs using the SPACE and ESPACE methods

SPACE			ESPACE		
No.	Gene	Degree	No.	Gene	Degree
1	PRC1	26	1	**AURKA**	36
2	RRM2	17	2	NKX2-1	35
3	GPR116	16	3	RRM2	18
4	NKX2-1	16	4	CYP2B7P1	16
5	CYP2B7P1	15	5	GPR116	16
6	SFTPB	15	6	SFTPB	15
7	HOP	13	7	HOP	12
8	C1orf116	12	8	HSD17B6	11
9	HSD17B6	12	9	PRC1	11
10	TFF1	12	10	TFF1	11
11	CD302	10	11	C1orf116	10
12	FMO5	10	12	CD302	10
13	TPX2	8	13	FMO5	10
			14	UBE2C	10
			15	**APC**	8
			16	**CDKN3**	8
			17	TPX2	8

Bold font denotes the genes only identified by the modified method

**Fig. 6:**
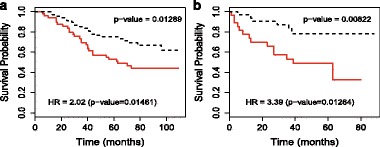
Kaplan-Meier curves for the CDKN3 gene from GSE13213 and GSE1037 datasets. For each gene, we divide patients into two groups, “High” and “Low”, by their gene expression levels with the K-means clustering method. *Red solid lines* denote the “High” group and *black dashed lines* denote the “Low” group. **a** CDKN3 (GSE13213 dataset) and **b** CDKN3 (GSE1037 dataset)

The CDKN3 (Cyclin-Dependent Kinase Inhibitor 3) protein coded by the CDKN3 gene is a cyclin-dependent kinase inhibitor. Recent studies [54, 55] show that CDKN3 overexpression was associated with poorer survival outcomes in lung adenocarcinoma, but not in lung squamous cell carcinoma. We validated that CDKN3 is associated with the prognosis of lung adenocarcinoma patients in two independent datasets (see Fig. 6). The CDKN3 expression allowed us to separate the lung adenocarcinoma patients into high CDKN3 and low CDKN3 groups with significantly different survival outcomes: in the GSE13213 dataset [56] (*n*=117), hazard ratio = 2.02 (high CDKN3 vs. low CDKN3), p=0.0146; in the GSE1037 dataset [57] (*n*=61), hazard ratio= 3.39 (high CDKN3 vs. low CDKN3), p=0.0126. Note that we divided patients into “high” and “low” groups by their gene expression levels with the K-means clustering method.

**Fig. 7:**
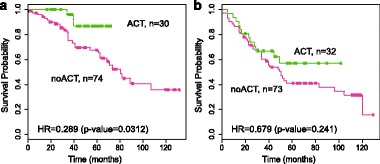
Kaplan-Meier curves for low and high groups of the AURKA gene expression in GSE42127 dataset [7]. The AURKA expression separates the 209 lung cancer patients into two groups. In the AURKA low expression group (*left panel*), lung cancer patients with ACT (*green line*) have significantly longer survival time than patients without ACT (observational group, *purple line*). In the AURKA high expression group (*right panel*), patients with ACT do not have significant survival benifit compared to patients without ACT. **a** lower expression group. **b** high expression group

APC (Adenomatous Polyposis Coli) is a tumor suppressor gene, and is involved in the Wnt signaling pathway as a negative regulator. It has been identified as one of the key mutation genes in lung adenocarcinoma by a comprehensive study on the somatic mutations in lung adenocarcinoma [58]. AURKA (aurora kinase A) is a protein-coding gene found to be associated with many different types of cancer. Aurora kinase inhibitors have been studied as a potential cancer treatment [59]. Using the GSE42127 dataset [7] (*n*=209), we found that AURKA expression can predict lung cancer patients’ response to chemotherapy. The dataset contains expression profiles and treatment information for 209 lung cancer patients from MD Anderson Cancer Center, among whom 62 received adjuvant chemotherapy (ACT group) and the remaining 147 did not (no ACT group). The AURKA gene expression allowed us to separate the 209 patients into a low AURKA group (*n*=104) and high AURKA group (*n*=105) using the median AURKA expression as a cut-off. The patients in the low AURKA group (Fig. 7 a) showed significant improvement in survival after ACT: hazard ratio = 0.289 (ACT vs. no ACT) and p value = 0.0312. The patients in the high AURKA group (Fig. 7 b), on the other hand, showed no significant survival benefit after ACT: hazard ratio = 0.679 (ACT vs. no ACT) and p value = 0.241. These results indicate that AURKA expression could potentially be a predictive biomarker for lung cancer adjuvant chemotherapy, since only patients with low AURKA expression benefit from the treatment, while those with high AURKA expression are less likely to benefit. In addition, it is possible that Aurora kinase inhibitors, which suppress the expression of AURKA genes, may synergize the effect of adjuvant chemotherary, i.e. improve the chance that a patient responds to adjuvant chemotherapy. In fact, a recent study has demonstrated that Aurora kinase inhibitors may synergize the effect of adjuvant chemotherapy in ovarian cancer, which is consistent with our results in lung cancer.

## Conclusions

We have demonstrated incorporating hub gene information in estimating network structures by extending SPACE with an additional tuning parameter. Our simulation study shows that the ESPACE method reduces errors in the construction of networks when the networks have previously-known hub nodes. Through two applications, we illustrate that the ESPACE method can improve the SPACE method by using the information about the potential hub genes. Although we adopted the GIC to select the optimal tuning parameters in this paper, the ESPACE method can directly be applied with other model selection criteria. The performance of the ESPACE method varies with the chosen criterion. However, the performance of the ESPACE method is at least comparable to the SPACE method since the ESPACE includes the SPACE as a reduced case.

## Abbreviations

ACT: Adjuvant chemotherapy
APC: Adenomatous polyposis coli
AURKA: Aurora kinase A
CDKN3: Cyclin-dependent kinase inhibitor 3
CMI2NI: Conditional mutual inclusive information-based network inference
*E.coli*: *Escherichia coli*
EGLASSO: Extended GLASSO
ESPACE: Extended SPACE
FDR: False discovery rate
GIC: Generalized information criterion
GGM: Gaussian graphical model
GLASSO: Graphical lasso
GLASSO-SF: GLASSO with reweighted strategy for scale-free network
GRN: Gene regulatory network
M3D: Many Microbe Microarrays database
MCC: Matthews correlation coefficients
MI: Mutual information
MISR: Mis-specification rate
MM: Minorization-maximization
NS: Neighborhood selection
PCACMI: path consistency algorithm based on conditional mutual information
PPI: Protein-protein interaction
SEN: Sensitivity
SPACE: Sparse partial correlation estimation
SPE: Specificity
TF: Transcription factor
